# SNMDA: A novel method for predicting microRNA‐disease associations based on sparse neighbourhood

**DOI:** 10.1111/jcmm.13799

**Published:** 2018-07-20

**Authors:** Yu Qu, Huaxiang Zhang, Cheng Liang, Pingjian Ding, Jiawei Luo

**Affiliations:** ^1^ School of Information Science and Engineering Shandong Normal University Jinan China; ^2^ School of Information Science and Engineering Hunan University Changsha China

**Keywords:** disease, miRNA, miRNA‐disease association, similarity measure, sparse neighbourhood

## Abstract

miRNAs are a class of small noncoding RNAs that are associated with a variety of complex biological processes. Increasing studies have shown that miRNAs have close relationships with many human diseases. The prediction of the associations between miRNAs and diseases has thus become a hot topic. Although traditional experimental methods are reliable, they could only identify a limited number of associations as they are time‐consuming and expensive. Consequently, great efforts have been made to effectively predict reliable disease‐related miRNAs based on computational methods. In this study, we present a novel approach to predict the potential microRNA‐disease associations based on sparse neighbourhood. Specifically, our method takes advantage of the sparsity of the miRNA‐disease association network and integrates the sparse information into the current similarity matrices for both miRNAs and diseases. To demonstrate the utility of our method, we applied global LOOCV, local LOOCV and five‐fold cross‐validation to evaluate our method, respectively. The corresponding AUCs are 0.936, 0.882 and 0.934. Three types of case studies on five common diseases further confirm the performance of our method in predicting unknown miRNA‐disease associations. Overall, results show that SNMDA can predict the potential associations between miRNAs and diseases effectively.

## 1 INTRODUCTION

miRNAs are a class of small noncoding RNAs which are associated with a variety of complex biological processes.^1^ Increasing studies have shown that miRNAs play a vital role in various biological processes which are essential for human life, including cell proliferation, differentiation, ageing and apoptosis.^2^, ^3^, ^4^, ^5^ In the meanwhile, evidence have demonstrated that miRNAs are related with a number of common neoplasms, such as breast neoplasms,^6^ lung neoplasms^7^ and prostate neoplasms.^8^ Therefore, the research on prediction of potential associations between miRNAs and diseases provides new opportunities to study the molecular mechanisms of diseases. During the past few years, a large number of associations have been confirmed by traditional experiments.^9^, ^10^ Although reliable, experimental methods are generally time‐consuming and expensive. As a result, effective computational methods are urgently needed to uncover potential associations between miRNAs and diseases.

Recently, a great number of computational methods have been proposed to identify miRNA‐disease associations. Under the assumption that miRNAs with similar functions are tend to be related with phenotypically similar diseases and vice versa,^11^ Jiang et al^12^ proposed the first computational model to predict miRNA‐disease associations by integrating the disease phenotype similarity network, miRNA functional similarity network and known phenome‐microRNAome network to build a heterogeneous network. Based on weighted K most similar neighbours, Xuan et al^13^ presented HDMP to predict the associations between miRNAs and diseases. The miRNA functional similarity was calculated by disease terms and the disease phenotype similarity. Considering the fact that the accuracy of local network similarity measures is lower than that of global network similarity measures,^14^, ^15^, ^16^, ^17^ Chen et al^18^ presented the first global method named RWRMDA by adopting random walk on the miRNA functional similarity network. However, RWRMDA cannot be applied to diseases without any known related miRNAs. Since then, many classical methods based on random walk were proposed to predict the latent miRNA‐disease associations. The main difference of these methods lies in the constructed networks on which the random walk was applied. Shi et al^19^ adopted random walk model to identify miRNA‐disease associations by integrating miRNA‐target relationship, disease‐genes in protein‐protein network. Later, Liu et al^20^ first calculated miRNA similarity based on the miRNA‐target and miRNA‐IncRNA associations, and they then constructed a heterogeneous network by integrating the semantic and functional similarities of disease, miRNA similarity and known miRNA‐disease associations. Similarly, Luo et al^21^ also constructed a heterogeneous network by integrating miRNA similarity, disease semantic similarity and known miRNA‐disease associations. However, they used an imbalanced birandom walk to identify miRNA‐related diseases. Chen et al^22^ proposed WBSMDA which integrates the Gaussian Interaction profile into the construction of similarity matrices. Specifically, WBSMDA calculated a within‐score and a between score, and combined them together to obtain a final score for miRNA‐disease associations prediction. Using the same data, they further proposed another method named HGIMDA.^23^ They first constructed a heterogeneous graph, and then implemented an iterative process on the graph to discover the relationships between miRNAs and diseases. HGIMDA was proved to be fast and effective compared to the aforementioned methods.

Recently, many path‐based methods were proposed to predict miRNA‐disease associations. Based on the lengths of different walks, the KATZ model was originally used in the social networks. Zou et al^24^ and Qu et al^25^ skillfully applied KATZ to predict the potential miRNA‐disease associations and achieved reliable results. You et al^26^ presented an effective method based on paths of different lengths. They constructed a heterogeneous network and applied depth‐first search algorithm to uncover the potential associations between miRNAs and disease. By taking the network topological structure into account, PBMDA achieved remarkable performance. Nevertheless, the searching process for paths of a certain length could be extremely time‐consuming in large networks. Recently, Chen et al^27^ proposed a method GIMDA to predict miRNA‐disease associations, in which the related score of a miRNA to a disease was calculated by measuring the graphlet interactions between two miRNAs or two diseases. They also^28^ proposed another model called NDAMDA based on network distance to predict miRNA‐disease associations. In this study, they considered not only the direct distance between two miRNAs (diseases), but also the average distance to other miRNAs (diseases).

Several machine learning‐based models were also developed to predict potential miRNA‐disease associations. Jiang et al^29^ trained a support vector machine classifier to distinguish positive miRNA‐disease associations from negative ones. Chen et al^30^ constructed a continuous classification function based on regularized least squares to reflect the probability of each miRNA related to a given disease. Subsequently, Luo et al^31^ presented an effective method KRLSM, which integrated different omics data and applied regularized least squares to discover the relationship between miRNAs and diseases. Recently, Chen et al^32^ proposed DRMDA using stacked auto‐encoder, greedy layer‐wise unsupervised pretraining algorithm and SVM to identify miRNA related with diseases. They also proposed another two machine learning‐based methods MKRMDA and EGBMMDA.^33^, ^34^ Specifically, MKRMDA could automatically optimize the combination of multiple kernels for disease and miRNA based on Kronecker regularized least squares. In EGBMMDA, a model of extreme gradient boosting machine was applied to identify miRNA‐disease associations. EGBMMDA achieved a high prediction accuracy in the framework of cross‐validation.

Although existing computational methods have made outstanding contributions in this filed, there is still room for further improvement. In this study, we present a reliable method based on Sparse Neighborhood to predict the MiRNA‐Disease Associations (SNMDA). SNMDA mainly consists of three steps. First, we use the sparse reconstruction to obtain the reconstructed similarity matrices both for miRNA and disease by considering the neighbourhood information. Second, we integrated similarity information to construct a similarity network for miRNAs and diseases, respectively. Third, we predicted the potential miRNA‐disease associations through label propagation^35^, ^36^, ^37^ on the miRNA similarity network as well as disease similarity network to obtain the final prediction result. To demonstrate the effectiveness of our method, we applied global LOOCV, local LOOCV and 5‐fold cross‐validation to evaluate the performance of our method, respectively. The corresponding AUCs are 0.936, 0.882 and 0.934, which in all cases outperform the four state‐of‐the‐art methods (HGIMDA,^23^ PBMDA,^26^ EGBMMDA 34 and MKRMDA^33^). Moreover, three types of case studies on five common neoplasms further validated the effectiveness of our method. Taken together, these results demonstrated that our method can effectively discover the underlying miRNA‐disease associations.

## 2 MATERIALS AND METHODS

### 2.1 Known miRNA‐disease associations

The human miRNA‐disease associations (HMDD)^38^ is a database containing experimentally verified miRNA‐disease associations. The known associations used in this paper were downloaded from the latest version HMDD v2.0 (http://www.cuilab.cn/hmdd). After filtering, 5430 associations between 383 diseases and 495 miRNAs were obtained. For convenience, we define an adjacency matrix *A* to describe the known miRNA‐disease associations. For a given disease *i* and a miRNA *j*,* A*(*i, j*) *=* 1 if *i* is related to *j*, and *A*(*i, j*) *=* 0 otherwise. Our goal is to confirm the uncertain associations between miRNAs and diseases.

### 2.2 miRNA functional similarity

According to previous study,^39^ the miRNA functional similarity was calculated based on the assumption that functionally similar miRNAs tend to be associated with similar diseases. Benefitting from their results, here we directly downloaded the miRNA functional similarity from http://www.cuilab.cn/files/images/cuilab/misim.zip. An adjacency matrix MFS was built to represent the similarity of miRNAs, where MFS(*i, j*) represents the similarity score between miRNA *i* and miRNA *j*. The larger the MFS(*i, j*) is, the closer their associations will be.

### 2.3 Disease semantic similarity

Mesh database provides a strict classification system for disease. Each disease can be described as a directed acyclic graph (DAG).^39^ DAG is made up of points and links. For a given disease *d*, DAG* =* (*d*,* T*(*d*), *E*(*d*)), where *T*(*d*) represents its ancestor nodes and itself, and *E*(*d*) is the set of links of *d*. Disease *t* is one of *T*(*d*), and the contribution to disease *d* can be calculated as follows^25^:

(1) $$\left\{\begin{array}{c} D_{d}\left(d\right)=1\\ D_{d}\left(t\right)=\max\left\{0.5*D_{d}\left(t^{\prime }\right)\left|t^{\prime }\in \text{child of}\;t\right.\right\}\text{if}\;t\neq d \end{array}\right.$$

We define the contribution to itself is 1 while others are 0.5. Therefore, we can use the following formula to calculate the semantic value of *d*.

(2) $$\text{DV}\left(d\right)=\sum_{T\in T_{d}}D_{d}\left(t\right)$$

Then, we calculate the semantic similarity between disease *a* and disease *b* through the following formula.

(3) $$S\left(a,b\right)=\frac{\sum_{T\in T_{a}\cap T_{b}}\left(D_{a}\left(t\right)+D_{b}\left(t\right)\right)}{\text{DV}\left(a\right)+\text{DV}\left(b\right)}$$

*D*_*d*_(*t*) represents the contribution of disease *t* to disease *a* while *D*_*b*_(*t*) represents the contribution of disease *t* to disease *b*. From Equation (3), we found that the semantic similarity for *a* and *b* depend on the number of their common diseases. The larger the number is, the greater the similarity will be. By calculating the semantic similarity of each disease pair according to Equation (3), we could obtain an adjacency matrix DSS. DSS(*i,j*) represents the semantic similarity between disease *i* and disease *j*.

### 2.4 SNMDA

**Figure 1:**
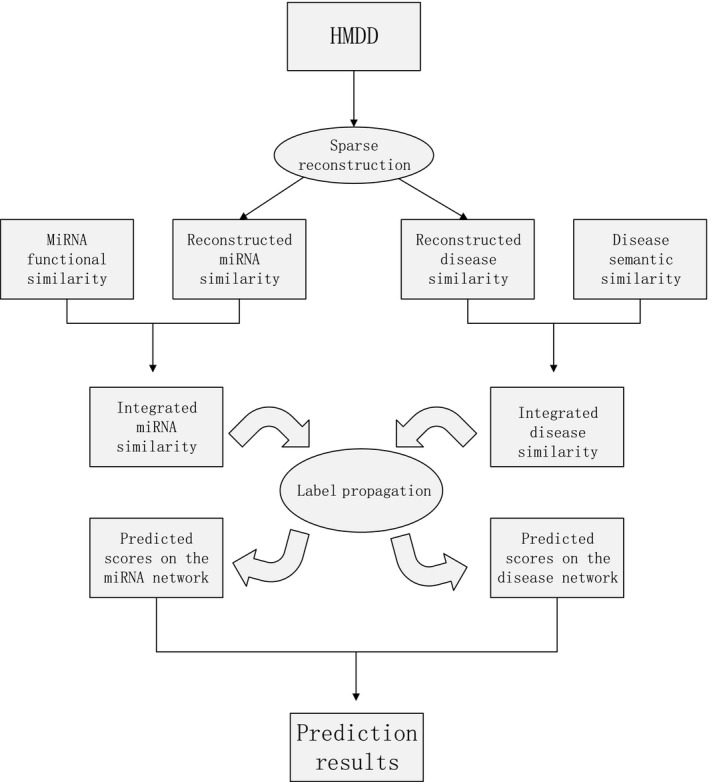
An overall workflow of SNMDA to predict miRNA‐disease associations

As described in the first section, SNMDA could be divided into three steps and the first step was the key to our approach. Specifically, we reconstructed miRNA similarity (RMS) and disease similarity (RDS) by taking the sparse neighbourhood into account. An overall workflow was illustrated in Figure 1. The details of SNMDA are as follows.

#### 2.4.1 Feature representation

**Figure 2:**
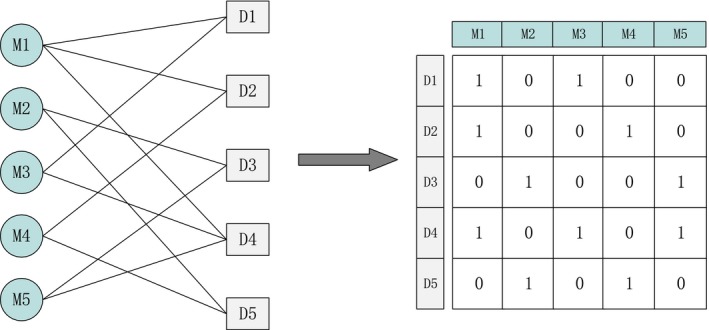
An example of feature representation

In general, the sparse neighbourhood representation is constructed based on feature vectors. Therefore, the miRNAs and diseases are required to be in the form of feature vectors. Here, “interaction profile”^36^, ^37^ is adopted to describe the feature vectors according to the known miRNA‐disease associations. An example is given in Figure 2.

As shown in Figure 2, the miRNA‐disease interaction network consists of 5 miRNAs and 5 diseases, where $\left\{\left.M1,M2,M3,M4,M5\right\}\right.$ and $\left\{\left.D1,D2,D3,D4,D5\right\}\right.$ represent the miRNA set and disease set, respectively. If miRNA *M1* is known to be related with disease *D*1, the value in the corresponding adjacency matrix of the interaction network is 1, and 0 otherwise. Each column represents the feature vector of one miRNA, and each row represents the feature vector of one disease. For example, the feature vector of *M*1 can be represented as (1, 1, 0, 1, 0) and that of *D*1 is represented as (1, 0, 1, 0, 0).

#### 2.4.2 Reconstruction of similarity for miRNAs and diseases

**Figure 3:**
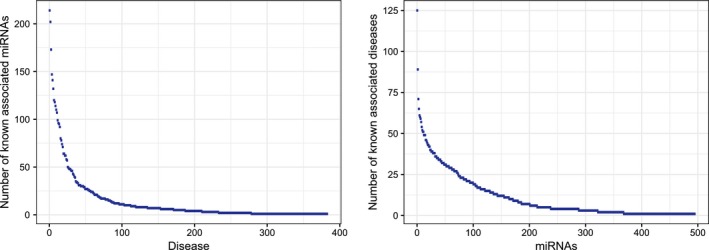
Degree distribution analysis for the constructed miRNA‐disease association network

Generally, the functional similarity of miRNAs as well as the semantic similarity of diseases is used to predict the relationships between miRNAs and diseases directly. However, they are still far from complete since many of the associations are uncovered yet. To solve this limitation, we first carried out a degree distribution analysis for the constructed miRNA‐disease association network (Figure 3). Obviously, power‐law distributions for both known disease‐associated miRNAs and known miRNA‐associated diseases were observed. In other words, most diseases as well as miRNAs only have very few known associations, which results in a very sparse heterogeneous network constructed by the known miRNA‐disease associations. Therefore, how to take advantage of this sparsity and further improve the prediction accuracy of the associations between miRNAs and diseases is a meaningful task.

In this section, we present a novel method to integrate the sparse information into the existing similarity information by reconstructing the miRNA similarity and disease similarity with sparse representation. Sparse representation has received extensive attention in pattern recognition and machine learning.^40^, ^41^, ^42^ Before calculating the reconstructed similarity, we first briefly introduce the definition of sparse neighbourhood and sparse reconstruction.

##### Sparse neighbourhood

The sparse neighbourhood of the sample *x*_*i*_ (*i = 1, 2,…, n*) is defined as follows. First, set a parameter *ε* (*ε >* 0) as the threshold. Then, compare the reconstructed coefficients with parameter *ε*. If reconstruction coefficient *α*_*j*_ > *ε*, (i≠j), we say *x*_*j*_ is one of the sparse neighbourhoods of samples *x*_*i*_. Otherwise, it does not belong to the sparse neighbourhoods of *x*_*i*_.^35^

##### Sparse reconstruction

Suppose we have an uncertain linear equation *x = D*α (*i = 1, 2,…, n*), where sample *x* is an *n*‐dimensional vector to be reconstructed, and *D* represents an over complete dictionary. α is a coefficient vector whose entries represent the correlation scores of sample *x* with other samples. Our motivation is to calculate the vector α. We can solve this problem by optimizing the following formula:

(4) $$\min_{\alpha }{\left||\alpha |\right|}_{0}\;\text{s.t.}x=D\alpha$$

L0‐norm is the number of nonzero elements in the vector. However, it is known as a NP‐hard problem to find the sparsest solution for L0‐norm. As L1‐norm is the closest convex form to L0‐norm,^43^ a common approach to solve this problem is to replace the L0‐norm with the L1‐norm.^43^ Consequently, the problem is equal to minimize the following optimizing problem:

(5) $$\min_{\alpha }{\left||\alpha |\right|}_{1}\text{s.t.}x=D\alpha$$

L1‐norm represents the sum of the absolute values of each element in a vector. *D* represents a dictionary. We can obtain a relevance score matrix by solving Equation (6). Considering that many scores in the matrix are very small, we only selected the sparse neighbourhood for each sample to reconstruct each sample by Equation (6).

(6) $$\min_{w}{\left||w|\right|}_{1}\text{s.t.}x=Dw$$

Eventually, we obtained a new matrix which is reconstructed by the sparse neighbourhood of each sample.

We have introduced sparse neighbourhood and sparse reconstruction. Next, we will introduce how to compute the reconstructed miRNA similarity and disease similarity. In the previous section, miRNAs and diseases have been represented as feature vectors and were regarded as points which were projected into a feature space, respectively. In our method, we assume that points are linearly arranged in the feature space, and every point can be reconstructed by other points.

#### 2.4.3 Integration of similarity information

After RDS and RMS were obtained, we integrated them into existing similarity matrices. The final miRNA similarity matrix (FMS) and final disease similarity matrix (FDS) were then used to predict the potential miRNA‐disease associations. Specifically, given a miRNA *x* and a miRNA *y*, if MFS(*x, y*)=0*,* then FMS(*x, y*) = RDS(*x, y*); otherwise, FMS(*x, y*) = (RMS(*x, y*) + MFS(*x, y*))/2. The FDS was calculated in the same way. The formulas are as follows:

(7) $$\text{FMS}\left(x,y\right)=\left\{\begin{array}{c} \text{RMS}\left(x,y\right),\;\text{if}\;\text{MFS}\left(x,y\right)=0\\ \frac{\text{RMS}\left(x,y\right)+\text{MFS}\left(x,y\right)}{2},\text{otherwise} \end{array}\right.$$

(8) $$\text{FDS}\left(x,y\right)=\left\{\begin{array}{c} \text{RDS}\left(x,y\right),\;\;\text{if}\;\text{DSS}\left(x,y\right)=0\\ \frac{\text{RDS}\left(x,y\right)+\text{DSS}\left(x,y\right)}{2},\text{otherwise} \end{array}\right.$$

According to Equation (7), we constructed a miRNA similarity network where nodes are miRNAs and edges represent their similarity. A disease similarity network was constructed in the same way according to Equation (8).

#### 2.4.4 Label propagation

Label propagation was applied on both miRNA and disease networks to obtain the prediction results. The labels were initiated with the known miRNA‐disease associations and were updated through label propagation. First, labels were propagated in the miRNA similarity network. In the process of label propagation, each point retains the information from its neighbours and receives its initial label information. Parameter *α* (0 < *α* < 1) is used to control the rate of retaining the information from its neighbours while 1‐*α* represents the probability of receiving its initial label information. Therefore, the iteration equation can be written as follows:

(9) $$F\left(t+1\right)=\alpha *\text{FMS}*F\left(t\right)+\left(1-\alpha \right)*Y$$

According to previous studies,^44^, ^45^ Equation (9) is guaranteed to converge if FMS is properly normalized by Equation (10):

(10) $$\text{MS}=D^{-1/2}*\text{FMS}*D^{1/2}$$

where *D* is a diagonal matrix with its (*i*,* i*)‐th element equal to the sum of *i*‐th row in FMS. We used the same way to deal with FDS. Therefore, Equation (9) was rewritten in the following form:

(11) $$F_{M}\left(t+1\right)=\alpha *\text{MS}*F_{M}\left(t\right)+\left(1-\alpha \right)*Y$$

Equation (11) was then used to update the label information for each miRNA until convergence. *F*_*M*_(*t +* 1) represents the label matrix in the (*t +* 1)‐*th* iteration. *Y* is the initial label matrix and *F*(0) *= Y*. When the iterative formula converges, *F*_*M*_(*t +* 1) was treated as the final correlation score matrix. Similarly, when labels are propagated in the disease similarity network, the iteration equation on the disease similarity network could be written as follows:

(12) $$F_{D}\left(t+1\right)=\alpha *\text{DS}*F_{D}\left(t\right)+\left(1-\alpha \right)*Y^{\prime }$$

Taken together, the final correlation score matrix is calculated by:

(13) $$F=\beta F_{M}+\left(1-\beta \right){F_{D}}^{\prime }$$

here, *β* was set to 0.5.

The complete process of SNMDA was outlined in Algorithm 1.

#### 2.4.5 Implementation details

SNMDA was implemented in MATLAB under the MATLAB R2016b programming environment. Specifically, the L1‐norm optimization problem was solved by the l1_ls MATLAB software package. All the experiments were performed on a desktop with an i7‐6700 3.40 GHz CPU and 16G RAM. The source code and data sets used in this work could be freely downloaded at https://github.com/misitequ/SNMDA.

## 3 RESULTS

### 3.1 Evaluation

We applied leave‐one‐out cross‐validation (LOOCV), and 5‐fold cross‐validation to test the prediction ability of our method. LOOCV can be conducted in two different ways: global and local LOOCV. In the framework of global LOOCV, each known miRNA‐disease association was left out in turn as the test sample and the other known associations were regarded as training samples.^46^ After each prediction, the ranking of the test sample was compared with all the unconfirmed miRNA‐disease associations. If the ranking of the test sample was higher than a given threshold, it was marked as a successful prediction. In comparison, in the framework of local LOOCV, each known miRNA associated with a given disease was left out in turn as the test sample and the ranking of that test sample was only compared with the unconfirmed associations of this specific disease.^26^ Both frameworks were repeated 5430 times. In addition, 5‐fold cross‐validation was also implemented to evaluate our method. In 5‐fold cross‐validation, all the known miRNA‐disease associations were divided into five disjoint subsets. Each subset was taken as test samples in turn while the rests were considered as training samples. To avoid the bias caused by sample divisions, we repeated 5‐fold cross‐validation 20 times and the final result is given by averaging the results from the 20 repetitions. Furthermore, the receiver operating characteristic (ROC) curves were plotted by calculating false‐positive rate (FPR) and true‐positive rate (TPR) at varying thresholds. The area under curve (AUC) was then calculated for comprehensive performance evaluations.

**Figure 4:**
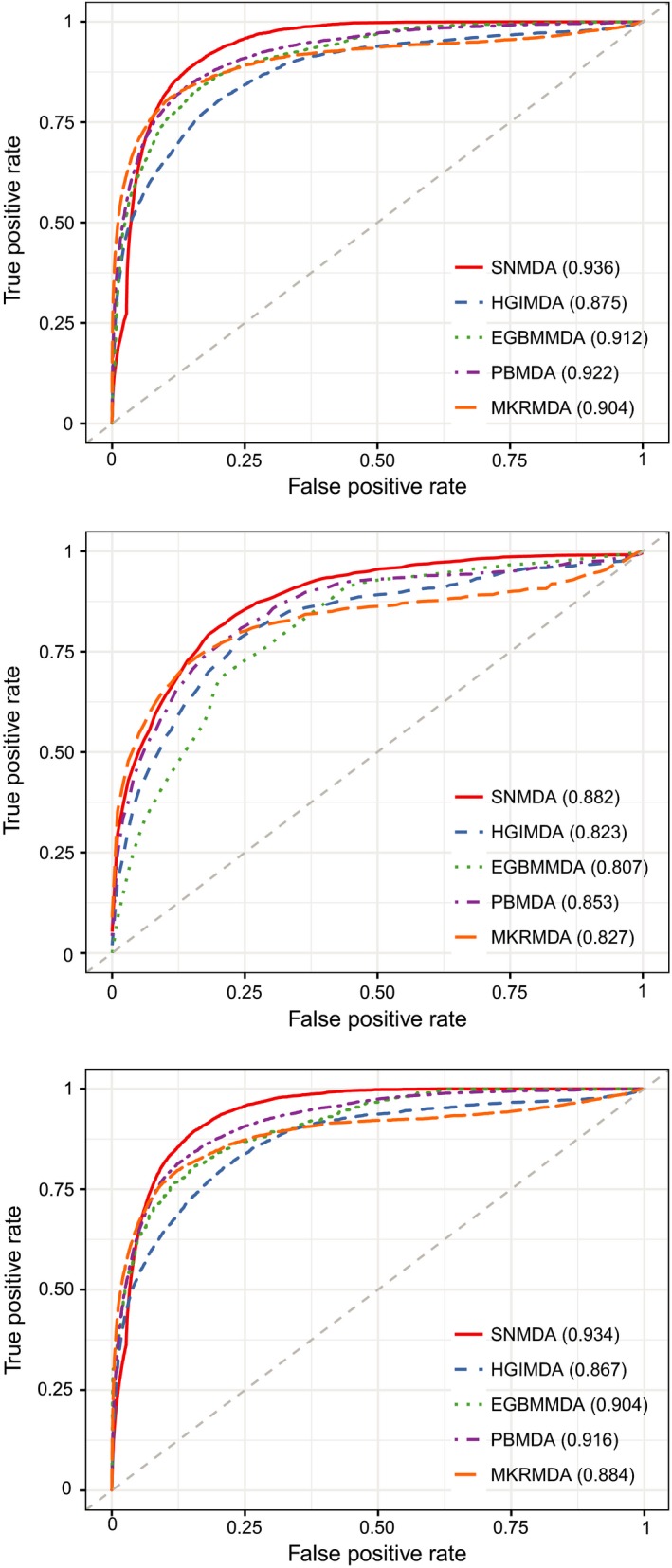
The results between SNMDA and the other methods (HGIMDA, EGBMMDA, PBMDA and MKRMDA) in terms of global LOOCV, local LOOCV and 5‐fold cross‐validation

As a result, SNMDA achieved reliable AUCs of 0.936, 0.882 and 0.934 for global LOOCV, local LOOCV and 5‐fold cross‐validation, respectively (Figure 4). To further prove the performance of SNMDA, we compared our method with four state‐of‐the‐art methods (HGIMDA,^23^ PBMDA,^26^ EBMMDA^34^ and MKRMDA^33^). As shown in Figure 4. In global LOOCV, HGIMDA, EBMMDA, PBMDA and MKRMDA achieved AUCs of 0.875, 0.922, 0.912 and 0.904, respectively, and the AUC obtained by SNMDA was higher than the other four methods by 0.061, 0.014, 0.024 and 0.032. In the framework of local LOOCV, the AUCs of HGIMDA, EBMMDA, PBMDA and MKRMDA were 0.823, 0.853, 0.807 and 0.827, all of which were lower than that of SNMDA. In five‐fold cross‐validation, the corresponding AUCs of the four methods were 0.867, 0.916, 0.904 and 0.88, respectively, where the performance of SNMDA consistently outperformed the other four alternatives. In conclusion, the cross‐validation results verified the superior ability of SNMDA in prediction the underlying associations between miRNAs and diseases.

### 3.2 Case study

In this section, we carried out three types of case studies to further validate the effectiveness of SNMDA. For the first type of case studies, we applied SNMDA to predict novel miRNA‐disease associations for three selected diseases based on the known associations from HMDD v2.0, that is breast neoplasms, lung neoplasms and prostate neoplasms. The prediction results for each disease were verified by two databases PhenomiR^47^ and dbDEMC,^48^ both of which provide differentially expressed miRNAs for certain diseases.

Breast neoplasms is a common disease in women and also a serious threat to the health of women.^49^, ^50^ In the early years, the mortality of breast neoplasms is just inferior to lung neoplasms. With the development of comprehensive treatment of breast neoplasms, the mortality rate has been significantly reduced. Researchers have found that many miRNAs are associated with breast neoplasms by clinical experiments, such as mir‐155 and mir‐21.^6^ We used our method to predict the candidate miRNAs for breast neoplasms, and we listed the top 50 predicted candidate miRNAs (Table 1). As a result, 49 of the top 50 candidate miRNAs were successfully verified. For example, hsa‐mir‐150 (1st in Table 1) and hsa‐mir‐130a are closely related to breast neoplasms. Hsa‐mir‐150 can promote human breast neoplasms growth^51^ while hsa‐mir‐130a (3rd in Table 1) could suppress breast neoplasms cell migration and invasion.^52^ The only unconfirmed miRNA was hsa‐mir‐507. As a matter of fact, Jia et al^53^ have reported that hsa‐mir‐507 inhibits the migration and invasion of human breast neoplasms cells. Our prediction results provided new evidence for its role in the pathogenesis of breast neoplasms.

**Table 1:** The top 50 predicted miRNAs associated with breast neoplasms

miRNA (1‐25)	Evidence	miRNA (26‐50)	Evidence
hsa‐mir‐150	dbDEMC; PhenomiR	hsa‐mir‐181d	dbDEMC; PhenomiR
hsa‐mir‐106a	dbDEMC; PhenomiR	hsa‐mir‐552	dbDEMC
hsa‐mir‐130a	dbDEMC; PhenomiR	hsa‐mir‐494	dbDEMC; PhenomiR
hsa‐mir‐192	dbDEMC; PhenomiR	hsa‐mir‐330	dbDEMC; PhenomiR
hsa‐mir‐15b	dbDEMC; PhenomiR	hsa‐mir‐198	dbDEMC; PhenomiR
hsa‐mir‐142	PhenomiR	hsa‐mir‐376a	dbDEMC; PhenomiR
hsa‐mir‐30e	PhenomiR	hsa‐mir‐211	dbDEMC; PhenomiR
hsa‐mir‐98	dbDEMC; PhenomiR	hsa‐mir‐1299	dbDEMC
hsa‐mir‐92b	dbDEMC	hsa‐mir‐455	PhenomiR
hsa‐mir‐372	dbDEMC; PhenomiR	hsa‐mir‐144	dbDEMC; PhenomiR
hsa‐mir‐151	dbDEMC; PhenomiR	hsa‐mir‐181c	dbDEMC; PhenomiR
hsa‐mir‐32	dbDEMC; PhenomiR	hsa‐mir‐381	dbDEMC; PhenomiR
hsa‐mir‐130b	dbDEMC; PhenomiR	hsa‐mir‐432	dbDEMC; PhenomiR
hsa‐mir‐99b	dbDEMC; PhenomiR	hsa‐mir‐660	dbDEMC
hsa‐mir‐28	dbDEMC; PhenomiR	hsa‐mir‐331	PhenomiR
hsa‐mir‐449b	dbDEMC	hsa‐mir‐363	dbDEMC; PhenomiR
hsa‐mir‐95	dbDEMC; PhenomiR	hsa‐mir‐382	dbDEMC
hsa‐mir‐186	dbDEMC; PhenomiR	hsa‐mir‐154	dbDEMC; PhenomiR
hsa‐mir‐196b	dbDEMC; PhenomiR	hsa‐mir‐663b	dbDEMC; PhenomiR
hsa‐mir‐449a	dbDEMC; PhenomiR	hsa‐mir‐484	dbDEMC; PhenomiR
hsa‐mir‐451	dbDEMC; PhenomiR	hsa‐mir‐520e	dbDEMC; PhenomiR
hsa‐mir‐491	PhenomiR	hsa‐mir‐575	dbDEMC
hsa‐mir‐99a	dbDEMC; PhenomiR	hsa‐mir‐663	dbDEMC; PhenomiR
hsa‐mir‐424	dbDEMC; PhenomiR	hsa‐mir‐507	unconfirmed
hsa‐mir‐212	dbDEMC; PhenomiR	hsa‐mir‐136	dbDEMC; PhenomiR

Lung neoplasms is a malignant tumour that has the greatest threat to the health and life for people. It is also one of the fastest growing neoplasms in the incidence and mortality rate.^7^ Increasing evidence has suggested that miRNAs can not only be utilized to classify lung cancer, but also has the potential to be biomarkers for early diagnosis and clinical treatments. As shown in the table (Table 2), 49 of the top 50 the predicted candidates were verified to be associated with lung neoplasms. For example, previous research demonstrated that hsa‐mir‐106a and hsa‐mir‐106b (2nd in Table 2) can affect the growth and metastasis of lung neoplasms. The result further proved that our method could effectively predict miRNA‐disease associations in lung neoplasms.

**Table 2:** The top 50 predicted miRNAs associated with lung neoplasms

miRNA (1‐25)	Evidence	miRNA (26‐50)	Evidence
hsa‐mir‐16	dbDEMC; PhenomiR	hsa‐mir‐193b	dbDEMC; PhenomiR
hsa‐mir‐106b	dbDEMC; PhenomiR	hsa‐mir‐302d	dbDEMC; PhenomiR
hsa‐mir‐429	dbDEMC; PhenomiR	hsa‐mir‐99b	dbDEMC; PhenomiR
hsa‐mir‐141	dbDEMC; PhenomiR	hsa‐mir‐28	dbDEMC; PhenomiR
hsa‐mir‐15a	dbDEMC; PhenomiR	hsa‐mir‐153	dbDEMC; PhenomiR
hsa‐mir‐20b	dbDEMC; PhenomiR	hsa‐mir‐488	dbDEMC; PhenomiR
hsa‐mir‐195	dbDEMC; PhenomiR	hsa‐mir‐10a	dbDEMC
hsa‐mir‐92b	dbDEMC; PhenomiR	hsa‐mir‐451	dbDEMC; PhenomiR
hsa‐mir‐15b	dbDEMC; PhenomiR	hsa‐mir‐129	dbDEMC; PhenomiR
hsa‐mir‐130a	dbDEMC; PhenomiR	hsa‐mir‐196b	dbDEMC; PhenomiR
hsa‐mir‐302c	dbDEMC; PhenomiR	hsa‐mir‐452	dbDEMC; PhenomiR
hsa‐mir‐302a	dbDEMC; PhenomiR	hsa‐mir‐299	PhenomiR
hsa‐mir‐296	PhenomiR	hsa‐mir‐383	dbDEMC; PhenomiR
hsa‐mir‐302b	dbDEMC; PhenomiR	hsa‐mir‐449a	dbDEMC; PhenomiR
hsa‐mir‐372	dbDEMC; PhenomiR	hsa‐mir‐516a	unconfirmed
hsa‐mir‐23b	dbDEMC; PhenomiR	hsa‐mir‐424	dbDEMC; PhenomiR
hsa‐mir‐194	dbDEMC; PhenomiR	hsa‐mir‐139	dbDEMC; PhenomiR
hsa‐mir‐373	dbDEMC; PhenomiR	hsa‐mir‐342	dbDEMC; PhenomiR
hsa‐mir‐520b	dbDEMC	hsa‐mir‐449b	dbDEMC; PhenomiR
hsa‐mir‐204	dbDEMC; PhenomiR	hsa‐mir‐99a	dbDEMC; PhenomiR
hsa‐mir‐339	dbDEMC; PhenomiR	hsa‐mir‐491	dbDEMC
hsa‐mir‐367	dbDEMC; PhenomiR	hsa‐mir‐181d	dbDEMC; PhenomiR
hsa‐mir‐215	dbDEMC; PhenomiR	hsa‐mir‐149	dbDEMC; PhenomiR
hsa‐mir‐130b	dbDEMC; PhenomiR	hsa‐mir‐340	dbDEMC; PhenomiR
hsa‐mir‐151	PhenomiR	hsa‐mir‐301b	dbDEMC

Prostate neoplasms is also one of the common diseases in men. It is estimated that one in six men in the United States will be diagnosed with prostate cancer in their lifetime, with the likelihood increasing with age.^54^ Studies have shown that miR‐182‐5p can be used as a marker for the diagnosis of prostate neoplasms and miR‐20 plays a vital role in the occurrence of prostate cancer.^55^ Therefore, identifying miRNAs related with prostate neoplasms is of great importance. As shown in Table 3, 48 candidate miRNAs were verified to be correctly related with prostate neoplasms. Only two miRNAs, hsa‐mir‐34c and hsa‐mir‐486, were not recorded in the two databases. As a matter of fact, studies have revealed that hsa‐mir‐34c together with hsa‐mir‐34a and hsa‐mir‐34b are the most frequently reported epigenetically dysregulated miRNAs.^56^ Our prediction was also in accordance with this result and provided new evidence for its association with prostate cancer. Besides, several studies have confirmed that hsa‐mir‐486 plays a important part in prostate cancer through negative regulation of multiple tumour suppressor pathways.^57^

**Table 3:** The top 50 predicted miRNAs associated with prostate neoplasms

miRNA (1‐25)	Evidence	miRNA (26‐50)	Evidence
hsa‐mir‐21	dbDEMC; PhenomiR	hsa‐mir‐223	dbDEMC; PhenomiR
hsa‐mir‐155	dbDEMC; PhenomiR	hsa‐mir‐29a	dbDEMC; PhenomiR
hsa‐mir‐17	dbDEMC; PhenomiR	hsa‐mir‐203	dbDEMC; PhenomiR
hsa‐mir‐146a	PhenomiR	hsa‐mir‐100	dbDEMC; PhenomiR
hsa‐mir‐20a	dbDEMC; PhenomiR	hsa‐mir‐29b	dbDEMC; PhenomiR
hsa‐mir‐34a	dbDEMC; PhenomiR	hsa‐mir‐31	dbDEMC; PhenomiR
hsa‐mir‐126	dbDEMC; PhenomiR	hsa‐mir‐146b	dbDEMC; PhenomiR
hsa‐mir‐92a	dbDEMC; PhenomiR	hsa‐let‐7d	dbDEMC; PhenomiR
hsa‐let‐7a	dbDEMC; PhenomiR	hsa‐mir‐29c	dbDEMC; PhenomiR
hsa‐mir‐200b	dbDEMC; PhenomiR	hsa‐mir‐205	dbDEMC; PhenomiR
hsa‐mir‐200c	dbDEMC; PhenomiR	hsa‐mir‐214	dbDEMC; PhenomiR
hsa‐mir‐143	dbDEMC; PhenomiR	hsa‐mir‐222	dbDEMC; PhenomiR
hsa‐mir‐200a	dbDEMC; PhenomiR	hsa‐mir‐182	dbDEMC; PhenomiR
hsa‐mir‐16	dbDEMC; PhenomiR	hsa‐mir‐133b	dbDEMC; PhenomiR
hsa‐mir‐221	dbDEMC; PhenomiR	hsa‐mir‐375	dbDEMC; PhenomiR
hsa‐mir‐1	dbDEMC; PhenomiR	hsa‐mir‐101	dbDEMC; PhenomiR
hsa‐mir‐18a	dbDEMC; PhenomiR	hsa‐mir‐34b	PhenomiR
hsa‐let‐7b	dbDEMC; PhenomiR	hsa‐mir‐210	dbDEMC; PhenomiR
hsa‐mir‐199a	dbDEMC; PhenomiR	hsa‐mir‐218	dbDEMC; PhenomiR
hsa‐mir‐15a	dbDEMC; PhenomiR	hsa‐mir‐9	PhenomiR
hsa‐mir‐141	dbDEMC; PhenomiR	hsa‐mir‐142	PhenomiR
hsa‐mir‐19a	dbDEMC; PhenomiR	hsa‐mir‐27a	dbDEMC; PhenomiR
hsa‐mir‐19b	dbDEMC; PhenomiR	hsa‐mir‐133a	dbDEMC; PhenomiR
hsa‐mir‐34c	unconfirmed	hsa‐let‐7e	dbDEMC; PhenomiR
hsa‐let‐7c	dbDEMC; PhenomiR	hsa‐mir‐486	unconfirmed

The second type of case study mainly aims to prove the ability of our method in predicting new associations for diseases without any known related miRNAs. To this end, we selected another disease pancreatic neoplasms for the following analysis. Pancreatic neoplasms is a malignant tumour of the digestive tract that is highly malignant and difficult to diagnose and treat in the world.^58^ Many studies have found that the differential expression of miRNAs in pancreatic neoplasms is closely related to the occurrence of tumours. Here, we first removed all known entries associated with pancreatic neoplasms in HMDD v2.0, and thus, all 495 miRNAs were considered as candidate miRNAs. Then, we used our model to prioritize these candidate miRNAs and obtained their corresponding rankings associated with pancreatic neoplasms. We found that all the top 50 predicted miRNAs were confirmed either by dbDEMC, PhenomiR or HMDD v2.0 (Table 4). The result proved that our method could be applied to predict potential associations for disease without any known related miRNAs.

**Table 4:** The top 50 predicted miRNAs associated with pancreatic neoplasms

miRNA (1‐25)	Evidence	miRNA (26‐50)	Evidence
hsa‐mir‐21	dbDEMC; PhenomiR;HMDD	hsa‐mir‐31	dbDEMC; PhenomiR;HMDD
hsa‐mir‐155	dbDEMC; PhenomiR;HMDD	hsa‐mir‐19b	dbDEMC; PhenomiR
hsa‐mir‐146a	dbDEMC; PhenomiR;HMDD	hsa‐mir‐19a	dbDEMC; PhenomiR
hsa‐mir‐125b	dbDEMC; PhenomiR;HMDD	hsa‐mir‐141	dbDEMC; PhenomiR
hsa‐mir‐145	dbDEMC; PhenomiR;HMDD	hsa‐mir‐195	dbDEMC; PhenomiR
hsa‐mir‐17	dbDEMC; PhenomiR;HMDD	hsa‐mir‐199a	dbDEMC; PhenomiR;HMDD
hsa‐mir‐34a	dbDEMC; PhenomiR;HMDD	hsa‐mir‐29c	dbDEMC; PhenomiR
hsa‐mir‐221	dbDEMC; PhenomiR;HMDD	hsa‐mir‐9	dbDEMC; PhenomiR
hsa‐mir‐16	dbDEMC; PhenomiR;HMDD	hsa‐let‐7c	dbDEMC; PhenomiR;HMDD
hsa‐mir‐126	dbDEMC; PhenomiR;HMDD	hsa‐mir‐210	dbDEMC; PhenomiR;HMDD
hsa‐mir‐20a	dbDEMC; PhenomiR;HMDD	hsa‐mir‐375	dbDEMC; PhenomiR;HMDD
hsa‐mir‐29a	dbDEMC; PhenomiR	hsa‐mir‐181a	dbDEMC; PhenomiR
hsa‐let‐7a	dbDEMC; PhenomiR;HMDD	hsa‐mir‐133b	dbDEMC; PhenomiR;HMDD
hsa‐mir‐200b	dbDEMC; PhenomiR;HMDD	hsa‐mir‐26a	dbDEMC; PhenomiR;HMDD
hsa‐mir‐29b	dbDEMC; PhenomiR;HMDD	hsa‐mir‐122	dbDEMC; PhenomiR;HMDD
hsa‐mir‐92a	dbDEMC; PhenomiR;HMDD	hsa‐mir‐133a	dbDEMC; PhenomiR
hsa‐mir‐222	dbDEMC; PhenomiR;HMDD	hsa‐mir‐34c	HMDD
hsa‐mir‐200c	dbDEMC; PhenomiR;HMDD	hsa‐let‐7d	dbDEMC; PhenomiR;HMDD
hsa‐mir‐1	dbDEMC; PhenomiR	hsa‐let‐7e	dbDEMC; PhenomiR;HMDD
hsa‐mir‐15a	dbDEMC; PhenomiR;HMDD	hsa‐mir‐182	dbDEMC; PhenomiR;HMDD
hsa‐mir‐200a	dbDEMC; PhenomiR;HMDD	hsa‐mir‐203	dbDEMC; PhenomiR;HMDD
hsa‐mir‐18a	dbDEMC; PhenomiR;HMDD	hsa‐mir‐142	HMDD
hsa‐let‐7b	dbDEMC; PhenomiR;HMDD	hsa‐let‐7i	dbDEMC; PhenomiR;HMDD
hsa‐mir‐143	dbDEMC; PhenomiR;HMDD	hsa‐mir‐30b	dbDEMC; PhenomiR
hsa‐mir‐223	dbDEMC; PhenomiR;HMDD	hsa‐let‐7f	dbDEMC; PhenomiR;HMDD

Finally, we conducted the last type of case study by taking the associations from older version of HMDD as input and test whether SNMDA could uncover those newly added associations in the latest version of HMDD. The older version of HMDD contained 1395 associations between 271 miRNAs and 137 diseases.^30^ Colorectal neoplasms were selected as the investigated disease. As a result, 49 of the top 50 predicted miRNAs have been confirmed to be related with colorectal neoplasms by HMDD 2.0, PhenomiR and dbDEMC (Table 5). The only unconfirmed miRNA was hsa‐mir‐205. However, studies have indicated that hsa‐mir‐205 is also associated with colorectal neoplasms.^59^ Taken together, all case studies have shown that SNMDA can effectively and reliably uncover the potential associations between miRNAs and diseases.

**Table 5:** The top 50 predicted miRNAs associated with colorectal neoplasms

miRNA (1‐25)	Evidence	miRNA (26‐50)	Evidence
hsa‐mir‐221	dbDEMC; PhenomiR; HMDD	hsa‐mir‐126	dbDEMC; PhenomiR; HMDD
hsa‐mir‐155	dbDEMC; PhenomiR; HMDD	hsa‐mir‐24	dbDEMC; PhenomiR
hsa‐let‐7a	dbDEMC; PhenomiR; HMDD	hsa‐mir‐199a	PhenomiR; HMDD
hsa‐mir‐19a	dbDEMC; PhenomiR; HMDD	hsa‐mir‐127	PhenomiR; HMDD
hsa‐mir‐222	dbDEMC; PhenomiR; HMDD	hsa‐mir‐106b	dbDEMC; PhenomiR
hsa‐let‐7b	dbDEMC; PhenomiR; HMDD	hsa‐mir‐30c	dbDEMC; PhenomiR
hsa‐mir‐19b	dbDEMC; PhenomiR; HMDD	hsa‐mir‐191	dbDEMC; PhenomiR
hsa‐let‐7e	dbDEMC; PhenomiR; HMDD	hsa‐mir‐15a	dbDEMC; PhenomiR
hsa‐let‐7d	dbDEMC; PhenomiR	hsa‐mir‐214	dbDEMC; PhenomiR
hsa‐mir‐146a	dbDEMC; PhenomiR; HMDD	hsa‐mir‐9	dbDEMC; PhenomiR; HMDD
hsa‐let‐7f	dbDEMC; PhenomiR	hsa‐mir‐146b	PhenomiR; HMDD
hsa‐let‐7c	dbDEMC; PhenomiR; HMDD	hsa‐mir‐101	dbDEMC; PhenomiR
hsa‐mir‐223	dbDEMC; PhenomiR	hsa‐mir‐125a	dbDEMC; PhenomiR
hsa‐mir‐200b	dbDEMC; PhenomiR; HMDD	hsa‐mir‐25	dbDEMC; PhenomiR; HMDD
hsa‐let‐7i	dbDEMC; PhenomiR	hsa‐mir‐29a	dbDEMC; PhenomiR; HMDD
hsa‐mir‐125b	dbDEMC; PhenomiR; HMDD	hsa‐mir‐20b	dbDEMC; PhenomiR
hsa‐mir‐34a	dbDEMC; PhenomiR; HMDD	hsa‐mir‐200a	dbDEMC; PhenomiR; HMDD
hsa‐let‐7 g	dbDEMC; PhenomiR	hsa‐mir‐150	dbDEMC; PhenomiR; HMDD
hsa‐mir‐92a	dbDEMC; PhenomiR; HMDD	hsa‐mir‐205	unconfuirmed
hsa‐mir‐29b	dbDEMC; PhenomiR	hsa‐mir‐34c	PhenomiR; HMDD
hsa‐mir‐16	dbDEMC; PhenomiR; HMDD	hsa‐mir‐133a	dbDEMC; PhenomiR; HMDD
hsa‐mir‐132	dbDEMC; PhenomiR	hsa‐mir‐107	dbDEMC; PhenomiR; HMDD
hsa‐mir‐141	dbDEMC; PhenomiR; HMDD	hsa‐mir‐32	dbDEMC; PhenomiR
hsa‐mir‐1	dbDEMC; PhenomiR; HMDD	hsa‐mir‐93	dbDEMC; PhenomiR; HMDD
hsa‐mir‐106a	dbDEMC; PhenomiR; HMDD	hsa‐mir‐429	dbDEMC; PhenomiR

## 4 DISCUSSION

As it is unrealistic to make a large scale of prediction based on traditional experimental methods, identifying the associations buried in miRNAs and diseases based on computational models is still a hot topic, and it remains a challenging task to discover such associations accurately and efficiently. Therefore, we proposed a novel method based on sparse neighbourhood to predict miRNA‐disease associations. First, sparse reconstruction was used to obtain a reconstructed miRNA similarity and a disease similarity. Second, similarity information was integrated to construct a similarity network for miRNA and disease, respectively. Finally, label propagation was applied on the miRNA similarity network as well as disease similarity network to obtain the relevance scores for each miRNA‐disease association. Besides, we applied global LOOCV, local LOOCV and 5‐fold cross‐validation to test the prediction performance and SNMDA achieved remarkable results in all the three cross‐validation frameworks. In addition, we compared SNMDA with four state‐of‐the‐art methods and the results demonstrated the superior performance of SNMDA. The prediction ability of our method was further verified by three types of case studies on five common diseases.

The success of our model could be mainly attributed to two reasons. First, the reconstructed miRNA similarities and disease similarities based on the sparse neighbourhood have greatly compensated for the incompleteness of current data sets due to the inherent sparsity of the constructed miRNA‐disease network. Second, the label propagation process on both miRNA and disease networks ensured that the known labels were reliably propagated according to the reconstructed similarities. Nonetheless, the performance of SNMDA was sensitive to the quality of the miRNA similarity network and disease similarity network. More data sources should be integrated into our model to further improve the prediction accuracy of our model. Moreover, we simply combined the reconstructed similarity matrices with the precalculated similarity matrices by adding them together with equal weights, which might be a suboptimal result for the overall similarity. In conclusion, we believe that SNMDA could serve as a powerful tool for the prediction of miRNA‐disease associations.

## CONFLICT OF INTEREST

The authors confirm that there are no conflict of interests.

## **ALGORITHM 1:** SNMDA algorithm

**Table:** 

**Input:** Matrices *Y* ^*n* × *m*^, MFS^*n* × *n*^, DSS^*m* × *m*^, parameter *α*,* β*
**Output:** Predicted associations matrix *F*.
1: For *i ←* 1 to *n* do
2: Select the sparse neighborhood for miRNA *i* by Equation (5).
3: End for
4: Obtain a reconstructed miRNA similarity matrix by Equation (6).
5: For *j ←* 1 to *m* do
6: Select the sparse neighborhood for disease *j* by Equation (5).
7: End for
8: Obtain a reconstructed disease similarity matrix by Equation (6).
9: Integrate similarity information by Equations (7) and (8).
10: Repeat
11: $F_{M}\left(t+1\right)=\alpha *MS*F_{M}\left(t\right)+\left(1-\alpha \right)*Y$
12: Until convergence
13: Repeat
14: $F_{D}\left(t+1\right)=\alpha *DS*F_{D}\left(t\right)+\left(1-\alpha \right)*Y^{\prime }$
15*:* Until convergence
16: $F=\beta F_{M}+\left(1-\beta \right){F_{D}}^{\prime }$
17: **Return** *F*
